# Recent progress in metabolomics for analyzing common infertility conditions that affect ovarian function

**DOI:** 10.1002/rmb2.12609

**Published:** 2024-09-30

**Authors:** Hiroshi Kobayashi, Shogo Imanaka

**Affiliations:** ^1^ Department of Gynecology and Reproductive Medicine Ms.Clinic MayOne Kashihara Japan; ^2^ Department of Obstetrics and Gynecology Nara Medical University Kashihara Japan

**Keywords:** glycolysis, infertility, lipid metabolism, metabolomics, oxidative phosphorylation

## Abstract

**Background:**

Numerous efforts have been undertaken to identify biomarkers associated with embryo and oocyte quality to improve the success rate of in vitro fertilization. Metabolomics has gained traction for its ability to detect dynamic biological changes in real time and provide comprehensive metabolite profiles. This review synthesizes the most recent findings on metabolomic analysis of follicular fluid (FF) in clinical conditions leading to infertility, with a focus on the dynamics of energy metabolism and oocyte quality, and discusses future research directions.

**Methods:**

A literature search was conducted without time constraints.

**Main findings:**

The metabolites present in FF originate from five primary pathways: glycolysis, oxidative phosphorylation, lipid metabolism and β‐oxidation, nucleic acid synthesis, and ketogenesis. Metabolomic profiling can broadly categorize infertile women into two groups: those with infertility due to aging and endometriosis, and those with infertility associated with polycystic ovarian syndrome and obesity. In the former group, glycolysis and lipid metabolism are upregulated to compensate for mitochondrial dysfunction, whereas the latter group exhibits the opposite trend. Assessing the levels of glucose, pyruvate, lactate, and plasmalogens in FF may be valuable for evaluating oocyte quality.

**Conclusion:**

Metabolomic analysis, particularly focusing on energy metabolism in FF, holds promise for predicting female reproductive outcomes.

## 1 INTRODUCTION

Infertility affects approximately one in seven couples of reproductive age worldwide.^1^, ^2^ Over the past two decades, there has been a notable increase in the use of assisted reproductive technology (ART).^3^ An analysis of a registry database from 2007 to 2015 in Japan revealed that the live birth rate per embryo transfer (ET) was 15.7%.^4^ Further analysis of the ART registry for 2015 demonstrated that 1 in 19.7 neonates was born through ART in Japan.^4^ In the United Kingdom, the overall pregnancy and live birth rates per ET in 2014 were 36.3% and 26.5%, respectively.^5^ In developed nations, a substantial number of older women are opting for in vitro fertilization (IVF), a trend that contributes to declining birth rates.^3^, ^4^ It is well established that infertility rates are higher among older women, with advanced age being a primary risk factor for diminished female reproductive capacity due to a decline in oocyte quantity and quality, which is associated with increased infertility.^6^ A fundamental aspect of the aging process is the increase in chromosomal segregation errors during meiotic divisions, coupled with metabolic imbalances caused by mitochondrial damage, as well as alterations in mitochondrial DNA (mtDNA) copy number and mutations.^6^, ^7^ Besides aging, various complications and environmental factors, such as endometriosis, polycystic ovary syndrome (PCOS), obesity, and smoking, negatively impact fertility.^8^

Extensive research has been conducted to improve the success rates of fertility treatments.^5^ Previous studies have suggested that several factors, including embryo score, treatment history, total dose of FSH, infertility cause, female age, height, and endometrial thickness, are associated with live birth rates following assisted reproduction.^5^ A significant body of basic research, focusing on genomic, epigenomic, transcriptomic, and proteomic studies, has aimed to identify biomarkers for oocyte and embryo quality.^9^, ^10^ These studies have identified several key genes and proteins.^11^ Communication between the oocyte and its surrounding cumulus cells is crucial for supplying the energy required for oocyte meiotic maturation.^11^ Comparative proteomic analyses have revealed that an imbalance between energy requirements and supply can lead to increased oxidative stress, mitochondrial damage, and reduced energy production, which can have adverse effects on oocytes.^12^ Disruptions in processes related to mitochondrial oxidative phosphorylation and energy metabolism have also been observed in the context of maternal aging and infertility due to endometriosis or PCOS, which may further impair oocyte quality.^13^ Understanding changes in gene and protein expression is crucial for monitoring molecular pathways and cellular activities, which may elucidate the fundamental mechanisms underlying infertility.^6^

The rise of novel “omics” fields, including metabolomics and lipidomics, is aiding in the discovery of new biomarkers for endometriosis or infertility.^10^ For example, follicular fluid (FF) and extracellular vesicles (EVs) from patients undergoing ART are abundant in low‐molecular‐weight metabolites that play a crucial role in oocyte maturation. Omics approaches that comprehensively investigate the abundance and composition of these metabolites have become an effective tool for investigating potential markers of oocyte quality and developmental competence, and successful fertilization.^14^, ^15^ Metabolomics offers the advantage of detecting the ever‐changing biological phenomena due to the relatively small number of metabolites and has therefore become prevalent in the field of reproductive medicine.^5^, ^16^ Metabolomic analysis is increasingly recognized as one of the valuable integrated multi‐omics strategies for assessing the precision of transcriptomics and proteomics studies.^17^, ^18^ Researchers have initiated studies to identify alterations in the expression of particular metabolites, including lipids and amino acids, within the FF of patients struggling with infertility.^19^ To date, it has been found that the amount and composition of metabolites in FFs collected from patients with various infertility disorders, such as endometriosis,^20^, ^21^ PCOS,^22^, ^23^, ^24^, ^25^ and diminished ovarian reserve (DOR),^26^ differ from those in healthy controls.^19^ Additionally, various metabolites linked to oocyte quality are proposed as potential biomarkers for ART success, including successful pregnancy, implantation, and delivery.^19^, ^27^, ^28^, ^29^, ^30^, ^31^ However, only a limited number of studies have comprehensively compared patients with different infertility conditions and metabolic pathways. This review explores the latest data on metabolomic analysis of FF in various clinical conditions leading to infertility, including aging, endometriosis, PCOS, and obesity. In particular, we concentrate on the comprehensive landscape of energy metabolism, follicular development and maturation, as well as oocyte quality, and suggest potential future avenues of research.

## 2 METHODS

### 2.1 Search strategy and selection criteria

**FIGURE 1:**
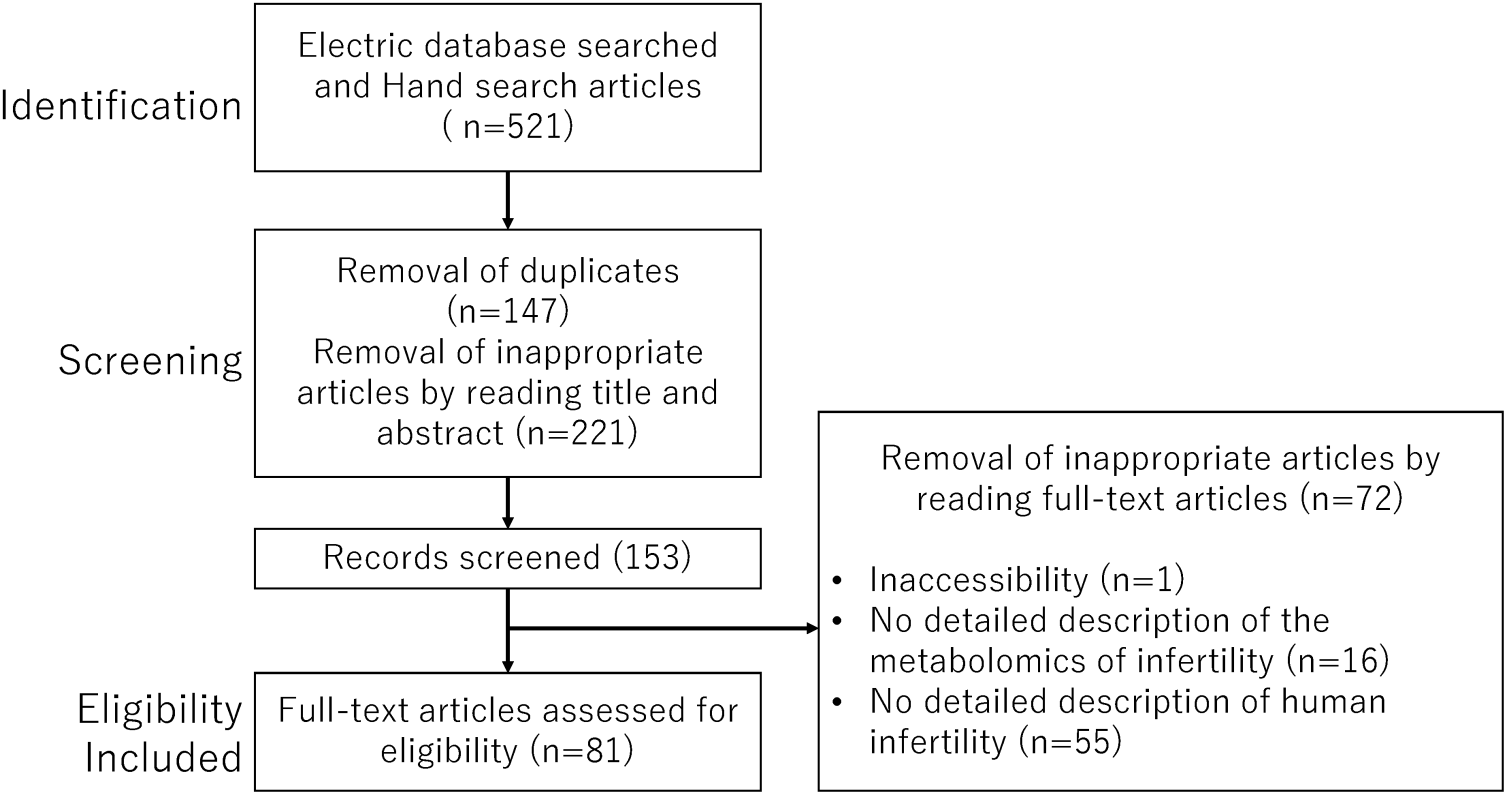
Flowchart depicting the study selection process.

This review conducted a comprehensive narrative analysis of the targeted literature focusing on metabolomics in endometriosis. Electronic databases, PubMed (https://pubmed.ncbi.nlm.nih.gov/) and Google Scholar (https://scholar.google.jp/), were searched for literature published up to May 1, 2024, using a combination of the following keywords: “metabolomics,” “infertility,” “follicular fluid,” “glycolysis,” “oxidative phosphorylation,” “lipid metabolism,” and “β‐oxidation.” The search terms were combined using Boolean operators, as outlined in Table 1. Additionally, a manual search of reference lists from published articles was performed. The studies included in this review were original research publications in English, along with reference lists from relevant review articles. Studies that were duplicated, irrelevant to the research topic, or published in non‐English languages were excluded. The study selection process is detailed in the flowchart depicted in Figure 1, which outlines the inclusion and exclusion criteria. The initial phase involved identifying records through electronic and manual searches. Titles and abstracts were subjected to preliminary screening, and after removing duplicates, non‐relevant studies were discarded. In the final phase of eligibility, full‐text articles were analyzed, and any from which detailed data could not be obtained were excluded. The authors independently evaluated the articles to determine their suitability for inclusion before reviewing the full texts.

**TABLE 1:** The keyword and search term combinations.

Search mode	The keyword and search term combinations
Search term 1	Metabolomics OR metabolite
Search term 2	Infertility OR assisted reproductive technology OR in vitro fertilization
Search term 3	Follicular fluid OR extracellular vesicle OR exosome
Search term 4	Glycolysis
Search term 5	Oxidative phosphorylation OR mitochondria
Search term 6	Lipid metabolism OR simple lipids OR complex lipids OR derived lipids
Search term 7	β‐oxidation OR fatty acids
Search	Search term 1 AND Search term 2
	Search term 1 AND Search term 2 AND Search term 3
	Search term 1 AND Search term 2 AND Search term 3 AND Search term 4
	Search term 1 AND Search term 2 AND Search term 3 AND Search term 5
	Search term 1 AND Search term 2 AND Search term 3 AND Search term 6
	Search term 1 AND Search term 2 AND Search term 3 AND Search term 7

## 3 RESULTS

### 3.1 Recent advancements in metabolomics in the field of female reproduction

Metabolomics, the study of functions and interactions of various metabolites such as amino acids, nucleotides, carbohydrates, lipids, and others, offers profound insights into the dynamic phenomena of reproductive processes.^5^, ^10^, ^32^, ^33^, ^34^ Comprehensive untargeted analysis of the human metabolome enables the examination of approximately 3000 metabolites.^5^ The first metabolomics investigation of FF for biomarkers of oocyte quality was reported in 2007.^35^ FF contains hormones, proteins, amino acids, enzymes, and fatty acids that influence the metabolism, development, and physiology of the oocyte.^10^, ^16^, ^36^, ^37^, ^38^, ^39^ Recent metabolomic methodologies involving the analysis of FF metabolites may enable the identification of infertility causes and the discovery of novel non‐invasive biomarkers.^3^, ^17^, ^39^, ^40^, ^41^, ^42^, ^43^, ^44^, ^45^ Furthermore, extracellular vesicles (EVs), such as exosomes, which carry RNAs, proteins, lipids, and amino acids, are crucial mediators of intercellular communication between various cell types (e.g., cumulus cells and oocytes), thereby influencing age‐related pathological conditions.^36^, ^46^, ^47^ We summarize current knowledge regarding metabolite alterations and evaluate whether these changes vary depending on the underlying condition.

### 3.2 Alterations in energy metabolism in various clinical conditions leading to infertility

**FIGURE 2:**
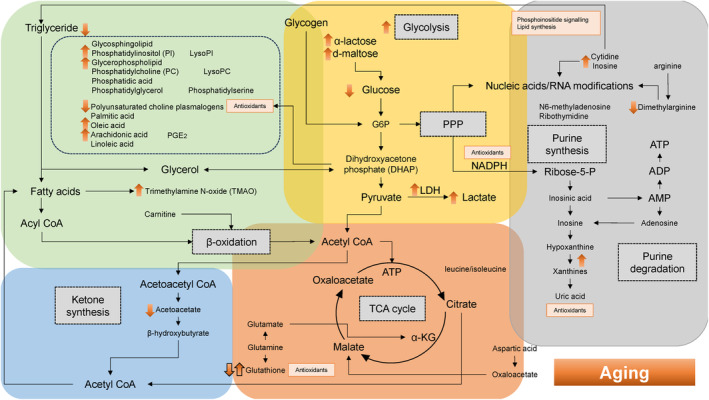
Dynamics of energy pathways in older women as determined by metabolomic analysis. The yellow, orange, green, blue, and gray boxes symbolize glycolysis, oxidative phosphorylation, lipid metabolism, ketogenesis, and nucleic acid metabolism, respectively. The metabolic‐level changes are represented by orange arrows.

**FIGURE 3:**
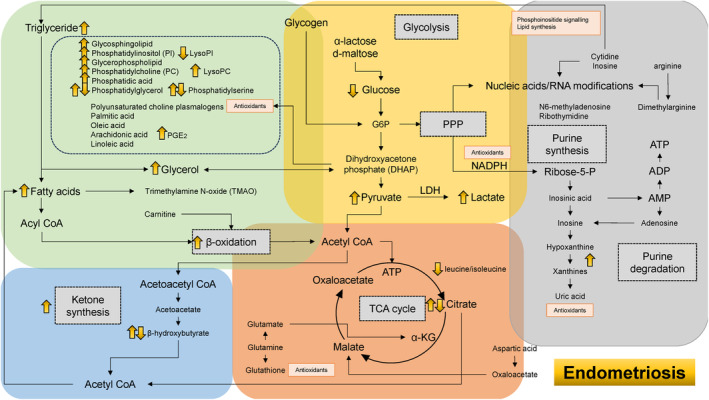
Dynamics of energy pathways in patients with endometriosis as determined by metabolomic analysis. The yellow, orange, green, blue, and gray boxes symbolize glycolysis, oxidative phosphorylation, lipid metabolism, ketogenesis, and nucleic acid metabolism, respectively. The metabolic‐level changes are represented by yellow arrows.

**FIGURE 4:**
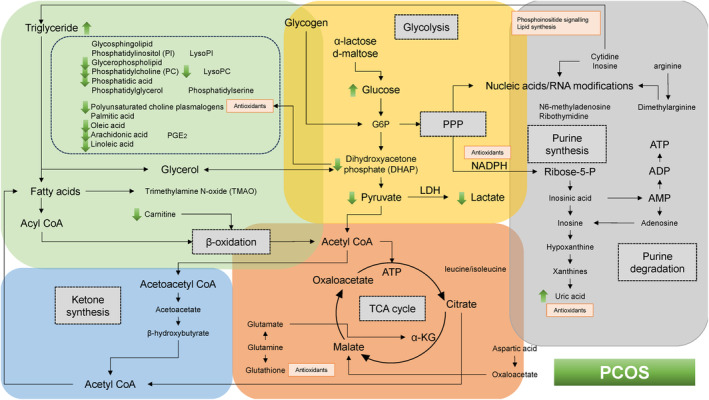
Dynamics of energy pathways in patients with PCOS as determined by metabolomic analysis. The yellow, orange, green, blue, and gray boxes symbolize glycolysis, oxidative phosphorylation, lipid metabolism, ketogenesis, and nucleic acid metabolism, respectively. The metabolic‐level changes are represented by green arrows.

**FIGURE 5:**
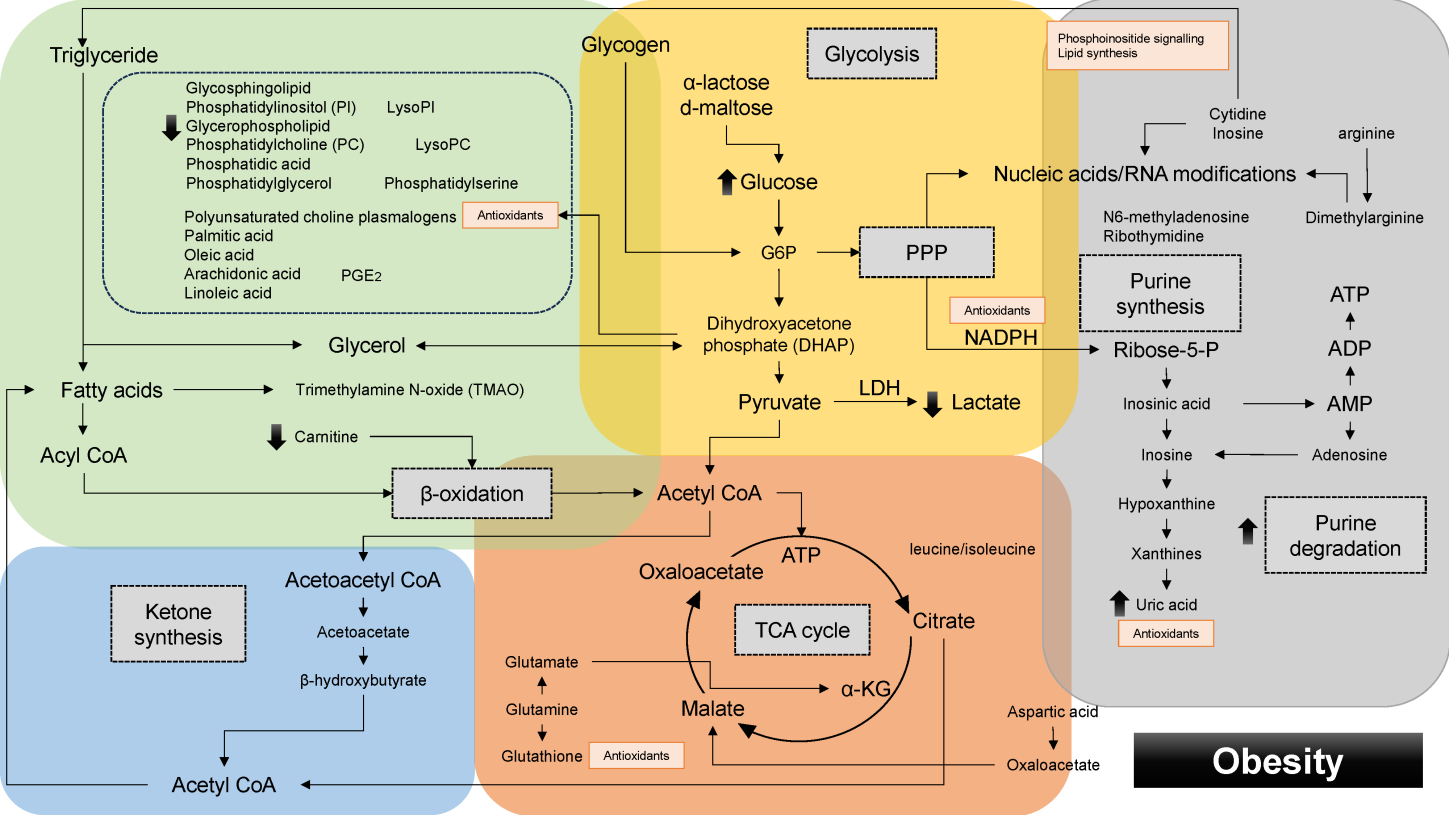
Dynamics of energy pathways in obese women as determined by metabolomic analysis. The yellow, orange, green, blue, and gray boxes symbolize glycolysis, oxidative phosphorylation, lipid metabolism, ketogenesis, and nucleic acid metabolism, respectively. The metabolic‐level changes are represented by black arrows.

Table 2 provides a summary of the references utilized in the construction of Figures 2, 3, 4, 5. This table includes the citations corresponding to each subsection from Sections 3.2.1, 3.2.5.

**TABLE 2:** Summary of the references cited for creating Figure 2. Among original articles, clinical research is highlighted with a yellow background, in vitro studies with green, and review articles with blue.

Original/review	Study design	Objectives and methods	Results	Ref. No.
Aging				
Original article	Clinical Research	Metabolomic analysis of the follicular fluid of 230 patients enrolled for the IVF cycle	↑Arachidonic acid, ↑ lyso‐PC(16:1), ↑ lyso‐PC(20:4), ↑ lyso‐PC(20:3), ↓lyso‐PC(18:3), and ↓ lyso‐PC(18:1)	48
	Clinical Research	Metabolomic analysis of the follicular fluid of 54 patients	↓ α‐Glucose, ↓ β‐glucose, ↑lactate, and ↑trimethylamine N‐oxide (TMAO) in patients above the age of 40	50
	Clinical Research	Metabolomic analysis of the follicular fluid of 54 patients enrolled for the IVF cycle	↓Glucose, ↑lactate, ↑progesterone, ↑glucose uptake, ↑lactate production, and ↑phosphofructokinase platelet	51
	Clinical Research	Metabolomic analysis of the follicular fluid of 21 patients enrolled for the IVF cycle	LDH activity increased with patient age and follicular size	52
	Clinical Research	Metabolomic analysis of the follicular fluid of 26 patients enrolled for the IVF cycle	↓Glucose, ↑lactate, and follicular fluid lactate concentration correlates negatively to glucose levels	54
	Clinical Research	Metabolomic analysis of the follicular fluid of 57 patients (28 diminished ovarian reserve patients, and 29 normal ovarian reserve patients) enrolled for the IVF cycle	DOR: Total dimethylarginine‐to‐arginine ratio, total polyunsaturated choline plasmalogens, and patient age	26
	Clinical Research	Metabolomic analysis of the follicular fluid extracellular vesicle of 30 patients enrolled for the IVF cycle	↑Total levels of the lipid classes in younger women	15
	Clinical Research	Metabolomic analysis of the follicular fluid of 29 patients enrolled for the IVF cycle	↑Phosphatidic acid, ↑phosphatidylinositol, ↑monogalactosyldiacylglycerol, ↑phosphatidylglycerol, ↑sphingomyelin, ↑diacylglycerol, and ↑triacylglycerol in the older group	55
	Clinical Research	Metabolomic analysis of the follicular fluid of 30 patients enrolled for the IVF cycle	Mitochondrial dysregulation in the advanced age group, with a modified balance between β‐oxidation and glycolysis	14
	Clinical Research	Metabolomic analysis of the follicular fluid of 54 patients enrolled for the IVF cycle	↓Linoleic acid and ↓eicosapentaenoic ethyl ester. Changes in composition and quantity: amino acids, indoles, nucleosides, organic acids, steroids, phospholipids, fatty acyls, and organic oxygen compounds	61
	Clinical Research	Metabolomic analysis of the follicular fluid of 58 women enrolled for the IVF cycle	Changes in composition and quantity: choline and phosphocholine	41
	Clinical Research	Metabolomic analysis of the follicular fluid of 62 women enrolled for the IVF cycle	The pregnancy cohort: ↑phosphatidic acid, ↑phosphatidylglycerol, ↑triacylglycerol, and ↓glucosylceramide. ↑Steroidogenesis, ↑cellular responses, ↑signal transduction, ↑cell cycle progression, ↑protein kinase C activation, and ↓apoptosis	3
	Clinical Research	Metabolomic analysis of the follicular fluid of 24 women enrolled for the IVF cycle	Oocytes that fertilized normally but failed to cleave:↑saturated fatty acids and↓polyunsaturated fatty acids	40
	Clinical Research	To examine the metabolomic profiling of follicular fluid‐derived exosomes from advanced‐age females	↑Alcohol [2‐hydroxypyridine], ↑amino acids [i.e., ratio of l‐glutamic acid/l‐glutamine, l‐threonine, beta‐alanine, and glutathione], ↑carbohydrates [alpha‐lactose, D‐maltose], ↑fatty acid [oleic acid], ↑nucleotides [inosine and cytidine], and ↑organic acids [the ratio of homogentisic acid/4‐hydroxyphenylpyruvic acid, taurine, and fumaric acid] in the advanced‐age group	36
	In vitro study (bovine)	To measure the activities of key enzymes involved in the regulation of glycolysis, the pentose phosphate pathway, and lipolysis in cumulus cells and the oocyte during in vitro maturation of bovine oocytes	In cumulus cells, phosphofructokinase was the most abundant followed by glucose‐6‐phosphate dehydrogenase and then lipase	53
	In vitro study (bovine)	To determine the effects of the three main fatty acids in follicular fluid (saturated palmitic acid, stearic acid, and unsaturated oleic acid) on mature oocytes	Palmitic and stearic acids inhibit bovine oocyte development, whereas oleic acid has the opposite effect, suggesting that the ratio and amount of saturated and unsaturated fatty acids are related to the developmental competence of maturing oocytes	58
	In vitro study (mouse)	To elucidate the mechanisms of fatty acid oxidation and meiotic resumption in oocytes	The activation of AMPK promotes the resumption of meiosis in oocytes by removing an impediment to fatty acid oxidation, indicating that reduced β‐oxidation may lead to impaired oocyte quality and meiotic arrest	62
	In vitro study (cattle)	The research examined how oleic acid impacts cell shape, programmed cell death, necrosis, growth, and hormone production in cultured granulosa cells	Increasing oleic acid concentrations induced intracellular lipid droplet accumulation, thus resulting in specific alterations in the morphology, gene expression, and steroid hormone production of cultured bovine granulosa cells	59
Review article		This review discusses the clinical implications of follicular fluid and cumulus cells for oocyte quality	Increased concentrations of superoxide dismutase (SOD), anti‐Mullerian hormone (AMH), lactate, and progesterone, and decreased concentrations of catalase and glucose were observed in older FF compared to younger FF	49
		To examine the studies that investigated the age‐related changes in cumulus cells and follicular fluid	Reproductive aging is characterized by a decrease in oocyte quantity and quality, which is associated with metabolomic alterations (heightened glycolytic activity and anaerobic metabolism), redox status imbalance, and increased apoptosis in the local oocyte microenvironment	6
		A review article on the structure and function of ether lipids such as plasmalogens	This review provides an overview of lipid types, structures, and functions to understand the function of ether lipids such as plasmalogens in follicular fluid	56
		A review article on energy and lipid metabolism in adipose tissue	Learning basic knowledge about energy and lipid metabolism in adipose tissue can help us understand the importance of decreased lipogenesis and increased lipolysis in follicular fluid in older women	57
		To understand the fundamentals of TMAO synthesis and metabolism, and to comprehend the role of TMAO in follicular fluid	This review addresses the synthesis of TMAO and its role in the progression of various inflammatory diseases, as well as its association with an increased risk of cardiovascular disease in patients with diabetes and obesity	60
		This review article on ceramide‐mediated apoptosis helps elucidate the intricate mechanisms of ceramide within follicular fluid	Ceramide, a class of sphingolipid, constitutes a fundamental component of cellular membranes. Under stress conditions, sphingomyelinase is activated, leading to the generation of ceramide through the hydrolysis of sphingomyelin. Ceramide, in turn, activates caspases and induces apoptosis	65
Endometriosis				
Original article	Clinical Research	Metabolomic analysis of the follicular fluid of 106 patients enrolled for the IVF cycle	Transcriptomic and metabolomic analysis. ↑Phosphatidylinositol and ↓lysophosphatidylinositol	63
	Clinical Research	The integration of different clinical experimental approaches	↑Phospholipids, ↑lactate, ↑insulin, ↑PTX3, ↑CXCL8, ↑CXCL10, ↑CCL11, ↑VEGF, ↓fatty acids, ↓lysine, ↓choline, ↓glucose, ↓aspartate, ↓alanine, ↓leucine, ↓valine, ↓proline, ↓phosphocholine, ↓total LDH, and ↓LDH‐3 isoform	65
	Clinical Research	Metabolomic analysis of follicular fluid from 20 women undergoing IVF cycles	↑Sphingolipids, ↑phosphatidylcholine, ↓phosphatidylglycerol phosphate, ↓phosphatidylserine, and ↓phosphatidylinositol diphosphate	20
	Clinical Research	Metabolomic analysis of follicular fluid from 33 women undergoing IVF cycles	↑LysoPC, ↑LysoPC, and ↓phytosphingosine	68
	Clinical Research	Metabolomic analysis of follicular fluid from 226 women undergoing IVF cycles	↑Lysophosphatidylcholine	69
	Clinical Research	Metabolomic analysis of follicular fluid from 49 women undergoing IVF cycles	↑Phosphatidic acid, ↑phosphatidylethanolamine, ↑phosphatidylcholine, ↑phosphatidylinositol, ↑glucosylceramide, and ↑1‐hydroxyvitamin D3 3‐D‐glucopyranoside	2
	Clinical Research	Metabolomic analysis of follicular fluid from 92 women undergoing IVF cycles	↑Sphingomyelin, ↑Phosphatidylcholine	72
	Clinical Research	To investigate whether phospholipase A2 (PLA2) activity is elevated in peritoneal fluid cells from women with endometriosis	↑PLA2 and ↑prostaglandins	73
	Clinical Research	Metabolomic analysis of follicular fluid from 79 women	↓Glucose, ↓citrate, ↓creatine, ↓tyrosine, ↓alanine, ↑lactate, ↑pyruvate, ↑lipids, and ↑ketone bodies in patients with deep invasive endometriosis. ↑Glycerol and ↑ketone bodies in patients with both deep invasive endometriosis and ovarian endometriosis	74
	In vitro study (mouse)	Mouse model of endometriosis	Transcriptomic and metabolomic analysis. ↑Phosphatidylinositol and ↓lysophosphatidylinositol	63
	In vitro study (mouse)	To explore whether the endogenous histone deacetylase inhibitor β‐hydroxybutyrate plays a role in mitigating oxidative stress	β‐Hydroxybutyrate suppresses oxidative stress by modulating the expression of oxidative stress resistance factors FOXO3A and MT2, as well as manganese superoxide dismutase and catalase	75
Review article		To synthesize findings on the identification of potential small molecule metabolite biomarkers in serum, ectopic tissue, eutopic endometrium, peritoneal fluid, follicular fluid, urine, cervical swabs, and endometrial fluid from patients with endometriosis	Glycerophospholipids may serve as potential biomarkers for the diagnosis of endometriosis	10
		To examine the latest findings on metabolome‐derived biomarkers in the follicular fluid of women with common infertility conditions (e.g., endometriosis, diminished ovarian reserve, and PCOS) and their potential impact on reproductive medicine outcomes	Reduced glucose concentrations and elevated levels of lactate and pyruvate were observed in the follicular fluid of patients with endometriosis and diminished ovarian reserve, while the opposite pattern was noted in patients with polycystic ovary syndrome	64
		To examine how lysophospholipids, specifically lysophosphatidic acid and sphingosine 1‐phosphate, modulate signaling, inflammation, and immune surveillance	Lysophospholipids have been associated with fibrosis, neuropathic pain, dysregulated angiogenesis, endometriosis, and cancer progression, establishing them as significant therapeutic targets	70
		To elucidate the functions of mammalian phospholipid biosynthetic enzymes	Disruption of genes encoding these enzymes has been demonstrated to cause embryonic lethality and significantly impact cell survival, proliferation, and anti‐apoptotic processes	71
PCOS				
Original article	Clinical Research	Metabolomic analysis of follicular fluid from 68 women undergoing IVF cycles	Alterations in vitamin B6 metabolism, phenylalanine metabolism, and carnitine biosynthesis as the predominant pathways affected in PCOS. 7β‐Hydroxycholesterol emerged as a potential biomarker for PCOS	22
	Clinical Research	Metabolomic analysis of follicular fluid from 18 women undergoing IVF cycles	Alterations in lipid subclasses, including triglycerides, phosphatidylethanolamine, and phosphatidylinositol	77
	Clinical Research	Metabolomic analysis of the follicular fluid of 58 women enrolled for the IVF cycle	↑Lactate	41
	Clinical Research	Metabolomic analysis of follicular fluid from 40 women undergoing IVF cycles	↑Free fatty acids (e.g., eicosapentaenoic acid), ↓bioactive lipids (e.g., LysoPC and phytosphingosine). ↑Phenylalanine, and↑leucine	24
	Clinical Research	Metabolomic analysis of follicular fluid and the waste embryo culture medium from 60 women undergoing IVF cycles	↓LysoPE, ↓L‐palmitoylcarnitine, ↓linoleylcarnitine, ↓arachidonoyl glycerophosphoinositol, and ↓LysoPA	13
	Clinical Research	Metabolomic analysis of follicular fluid and the waste embryo culture medium from 45 women undergoing IVF cycles	↓Plasmalogens	80
	Clinical Research	Analysis of venous blood samples from 57 women	↑Dehydroepiandrosterone sulfate, ↑total testosterone, ↑free androgen index, ↑luteinizing hormone, ↑low‐density lipoprotein cholesterol, ↑non‐high‐density lipoprotein cholesterol, ↑fasting insulin levels, ↑HOMA‐IR measurements, ↑LH/FSH ratios, ↓L‐carnitine, and↓total sex hormone‐binding globulin	82
	Clinical Research	Metabolomic analysis of follicular fluid from 20 women undergoing IVF cycles	Distinct metabolic pathways: lipid metabolism, carnitine metabolism, androgen metabolism, and bile acid metabolism	83
	In vitro study (human)	Granulosa cells collected during IVF cycles from seven women with normal ovaries, six ovulatory women with polycystic ovaries (ovPCO), and seven anovulatory women with polycystic ovaries (anovPCO)	Insulin‐dependent lactate production was significantly impaired in granulosa cells from anovPCO compared to those from either normal or ovPCO groups	78
Review article		To examine the latest findings on metabolome‐derived biomarkers in the follicular fluid of women with common infertility conditions (e.g., endometriosis, diminished ovarian reserve, and PCOS) and their potential impact on reproductive medicine outcomes	Reduced glucose concentrations and elevated levels of lactate and pyruvate were observed in the follicular fluid of patients with endometriosis and diminished ovarian reserve, while the opposite pattern was noted in patients with polycystic ovary syndrome	64
		To address biomarkers of oocyte and embryo quality, as well as biomarkers of IVF outcomes in embryo culture medium, follicular fluid, and blood plasma in female mammals	A metabolomic study is currently being conducted using human plasma to identify women with PCOS	17
		To offer an updated overview of the structures and physiological roles of plasmalogens, aiming to enhance our understanding of reproductive physiology	Plasmalogens may contribute to the development of more effective diagnostic and prognostic biomarkers for metabolic diseases such as obesity, diabetes, inflammation, neurodegeneration, and cancer	81
Obesity				
Original article	Clinical Research	Metabolomic analysis of follicular fluid and serum from 17 women undergoing IVF cycles	↑Uric acid, ↑isothreonic acid, ↑complex lipids, ↓2‐ketoglucose dimethylacetal, ↓aminomalonate, ↓complex lipids, and ↓indole‐3‐propionic acid (IPA)	84
	Clinical Research	Metabolomic analysis of follicular fluid from 160 women undergoing IVF cycles	↑Ganoderiol H, ↑LPI, ↑sedoheptulose ↑1,7‐bisphosphate, ↑austalide L, ↑2‐{[hydroxyl (3‐hydroxy‐4‐methoxyphenylmethylidene] amino} acetic acid, ↓1‐phenyl‐1,3‐eicosanedione, ↓retinol acetate, ↓p‐cresol sulfate, ↓setariol, and ↓arachidonyl carnitine	86
	In vitro study (equine)	Twenty mares were categorized into three groups: normal weight, obese, and obese diet supplemented. Granulosa cells, follicular fluid, and cumulus–oocyte complexes were collected	Obesity induced mitochondrial metabolic disturbances in granulosa cells, reduced L‐carnitine availability within the follicle, and promoted lipid accumulation in cumulus cells and oocytes. Obesity disrupts the equine ovarian follicle by promoting lipid accumulation and impairing mitochondrial function	87
Review article		To provide an update on the impact of obesity on human oocyte quality and development	Obesity triggers a range of biochemical alterations that negatively impact oocyte quality, particularly through hormonal imbalances, elevated oxidative stress, lipid metabolism abnormalities, and heightened inflammatory responses	85
Clinical Conditions Leading to Infertility				
Original article	Clinical Research	Metabolomic analysis of follicular fluid from 53 women with tubal diseases, unexplained infertility, endometriosis, and PCOS undergoing IVF cycles	Metabolomic profiling may differentiate PCOS and endometriosis from controls and predict oocyte quality	76
	Clinical Research	To quantify the concentrations of 55 water‐ and lipid‐soluble low‐molecular‐weight compounds in follicular fluid from 35 control participants and 145 infertile participants	Altered expression: antioxidants, oxidative/nitrosative stress‐related compounds, purines, pyrimidines, energy‐related metabolites, and amino acids	88
	Clinical Research	Metabolomic analysis of follicular fluid from women undergoing IVF cycles	Metabolites identified in the infertility group: phosphatidic acid, phosphatidylethanolamine, phosphatidylcholine, phosphatidylinositol, glucosylceramide, and 1‐hydroxyvitamin D3 3‐D‐glucopyranoside	2
Review article		To offer an updated perspective on the biological roles of hypoxanthine, xanthine, and uridin	As indicators of ATP depletion, hypoxanthine, xanthine, and uridine hold significant implications in reproductive pathophysiology	89

#### 3.2.1 Advanced reproductive age

In Figures 2, 3, 4, 5, the yellow box indicates glycolysis, the orange box signifies oxidative phosphorylation, the green box denotes lipid metabolism, the gray box corresponds to purine metabolism, and the blue box represents ketone body synthesis. Oocyte quality is known to decline with advancing age. Researchers have explored the relationship between age and reproductive function by examining differences in metabolites within the FF of patients of varying ages undergoing IVF and ET.^48^ In Figure 2, the orange upward and downward arrows indicate age‐related changes in metabolite levels. Patients were divided into younger and older groups, with advanced age typically defined as 35 or 40 years or older. Compared to younger women under 35 with normal ovarian reserves, older women and those with DOR exhibit lower glucose and higher lactate concentrations in their FF.^49^ Similar results were observed in women over 40 years of age.^50^ This may reflect an age‐related increase in the expression of glycolytic enzymes, such as phosphofructokinase^51^ and LDH.^52^ Phosphofructokinase was also abundant in bovine cumulus cells.^53^ Excessively low glucose levels in FF negatively impact oocyte maturation, necessitating enhanced glycogenolysis and the compensatory conversion of α‐lactose and d‐maltose into glucose.^36^ Indeed, analysis of exosomal metabolites in FF revealed higher levels of lactose and maltose in the older group compared to the younger group.^36^ Cumulus cells in women of advanced reproductive age must supply energy to the oocyte through further heightened glycolytic activity and anaerobic metabolism.^6^, ^54^ However, the availability of nutrients like pyruvate in the TCA cycle diminishes in the oocytes of older women due to mitochondrial dysfunction and impaired DNA repair mechanisms, leading to lactate accumulation. The increase in lactate levels results in a lowered pH of the FF, further impairing the quality of cumulus cells and oocytes.^50^ Thus, glycolysis and mitochondrial oxidative phosphorylation might be compromised in the cumulus cells and oocytes of older women. To counteract follicular stressors such as reduced pH, hypoxia, and elevated reactive oxygen species (ROS), and to meet the oocytes' demands in aging individuals, a compensatory upregulation of oxidative phosphorylation is necessary to boost ATP production.^6^

Additionally, aging is known to affect nucleic acid synthesis and metabolism. Higher levels of cytidine and inosine have been noted in older women compared to younger women, potentially contributing to enhanced nucleic acid synthesis via the upregulation of glycolysis and the pentose phosphate pathway (PPP) (Figure 2, gray square).^36^ The elevated levels of cytidine and inosine may indicate an upregulation of nucleic acid synthesis, alongside enhanced phosphoinositide signaling and lipid synthesis, likely as a compensatory mechanism.^36^

Furthermore, it has been reported that the dimethylarginine/arginine ratio is significantly decreased in the FF of older women with DOR (Figure 2, gray square).^26^, ^36^ Dimethylarginine functions as an inhibitor of nitric oxide synthase (NOS), thereby suppressing the production of nitric oxide, which is crucial for vasodilation and blood flow regulation. Thus, a decrease in dimethylarginine levels may suggest a loss of compensatory reserve, including anti‐inflammatory and vasodilatory effects.

Next, age‐related changes influence not only metabolites related to glycolysis, the PPP, and mitochondrial oxidative phosphorylation but also alter the lipid profile of FF.^48^ Women under 35 exhibit higher levels of lipids in FF or extracellular vesicles (EVs) compared to older women.^15^ Research indicates a decline in polyunsaturated choline plasmalogen, a complex lipid with antioxidant properties, with advancing age.^48^ This reduction is accompanied by a compensatory increase in lipid metabolism in women over 35, including glycosphingolipid, phosphatidylinositol phosphate, and glycerophospholipid metabolism,^6^, ^55^ as well as arachidonic and oleic acids (Figure 2, green square).^48^ Polyunsaturated choline plasmalogen has antioxidant properties and serves as a reservoir for polyunsaturated fatty acids, such as arachidonic acid and docosahexaenoic acid (DHA).^56^ Consequently, the diminished antioxidant capacity of plasmalogen leads to the accumulation of oxidized substances due to oxidative stress. Additionally, excess arachidonic acid heightens vascular permeability through the production of prostaglandins and leukotrienes, triggering inflammatory responses. Collectively, lower lipogenesis and higher lipolysis in older women may alter the availability of lipid metabolites, leading to decreased plasmalogen levels and increased unsaturated fatty acid concentrations, potentially impairing oocyte maturation.^14^, ^57^ Meanwhile, oleic acid has been reported to positively impact bovine oocyte maturation and subsequent embryo development, mitigating the harmful effects of saturated fatty acids such as palmitic and stearic acids.^36^, ^58^ Increased concentrations of oleic acid in the FF of older women may protect oocytes from damage due to reduced lipid storage.^36^, ^59^

Furthermore, an increase in trimethylamine N‐oxide (TMAO) levels and a concomitant decrease in acetoacetate levels have been observed in patients of advanced age (Figure 2, green square).^50^ TMAO is produced by the gut microbiota.^60^ Phosphatidylcholine (PC) undergoes metabolism to produce trimethylamine, which is subsequently converted to TMAO. Elevated TMAO levels suggest a reduced conversion of fatty acids to acyl‐CoA and a concomitant inhibition of β‐oxidation. Moreover, a decline in β‐oxidation results in lower acetoacetate levels due to the suppression of ketone synthesis (Figure 2, blue square). Diminished β‐oxidation leads to a reduction in ATP availability for oocytes, resulting in compromised oocyte quality and meiotic arrest.^61^, ^62^ Thus, increased levels of TMAO and reduced levels of acetoacetate could potentially serve as indicators of oocyte quality in older women.

Collectively, the lack of balance and coordination among glycolysis, oxidative phosphorylation, lipid metabolism, β‐oxidation, and nucleic acid synthesis with aging may contribute to the low success rates of IVF treatment. Fat burning could be accelerated in older women. However, the extent of glycolysis and lipid metabolism varies significantly depending on whether compensatory mechanisms remain intact or have been compromised. It is believed that factors influencing compensatory mechanisms include age‐related alterations in the expression of genes involved in various energy metabolism pathways, although the specifics remain unclear.

#### 3.2.2 Endometriosis

The yellow arrows in Figure 3 highlight the metabolites that are altered in patients with endometriosis. Consistent findings have been reported across multiple studies regarding glycolysis (e.g., glucose, pyruvate, and lactate) and fatty acid metabolism (e.g., glycerophospholipids and fatty acids) in endometriosis.^10^, ^63^ Patients with endometriosis exhibit decreased glucose levels and increased lactate and pyruvate concentrations in FF, indicative of enhanced anaerobic glycolysis.^64^, ^65^ The increased anaerobic glycolysis is associated with the Warburg effect, which mitigates oxidative stress by reducing oxidative phosphorylation and lipid peroxidation.^10^, ^66^ Moreover, phospholipid concentrations in the FF of endometriosis patients were elevated compared to controls,^65^ a finding that contrasts with observations in older women. Specifically, the metabolic profile in FF from women with endometriosis includes increased levels of glycerophospholipids, phosphatidyl serine (PS),^10^ PC,^20^ sphingolipids,^20^, ^67^ lysophosphatidylcholine (LPC),^10^, ^68^ and phosphatidylinositol‐4,5‐bisphosphate.^20^ For instance, the increase in LPC levels was particularly notable in large follicles compared to small follicles.^69^ The accumulation of phospholipids in endometriosis may be linked to the induction of oxidative stress and inflammatory processes.^2^, ^70^ Two significant classes of complex phospholipids, PC and sphingomyelin (SM), are also involved in cell proliferation, anti‐apoptosis, and processes of denervation and re‐innervation.^20^, ^71^, ^72^ Phosphatidylinositol diphosphate (PI diphosphate) generates the production of two second messenger molecules, inositol 1,4,5‐triphosphate (IP3) and diacylglycerol (DAG).^20^ Additionally, increased PI and decreased lysophosphatidylinositol (LPI) have been identified in follicles of patients with endometriosis‐associated infertility.^63^ Indeed, the proper balance of PI and LPI correlates with the number of retrieved and mature oocytes.^63^ Since LPI inhibits apoptosis and promotes cell proliferation, a decrease in LPI may lead to cell death.^63^ Given that PI is hydrolyzed by phospholipase A2 to generate LPI, phospholipase A2 expression may be reduced in endometriosis.^63^ However, phospholipase A2 activity is known to be elevated in peritoneal fluid cells of women with endometriosis.^73^ The decreased LPI levels may reflect a breakdown in compensatory mechanisms.

Furthermore, lipid, glycerol, and ketone body concentrations were elevated in women with deep infiltrating endometriosis (DIE) compared to control participants.^74^ These concentrations were further elevated in DIE patients with ovarian endometrioma.^74^ Increased glycerol levels lead to the activation of glycolysis via recruitment of the intermediate dihydroxyacetone phosphate (DHAP). Additionally, ketone synthesis is promoted by fatty acid degradation and β‐oxidation, resulting in elevated levels of β‐hydroxybutyrate. β‐Hydroxybutyrate also enhances antioxidant capacity by inducing the expression of manganese superoxide dismutase and catalase.^75^ However, sustained upregulation of energy metabolism through fatty acid β‐oxidation may lead to a decrease in certain phospholipid species over time.^10^ Since mitochondria are involved in pyruvate oxidation, the TCA cycle, oxidative phosphorylation, fatty acid oxidation, and ketogenesis, compensatory mitochondrial function may result in increased oxidative phosphorylation, fatty acid synthesis, and β‐oxidation, but decreased ketogenesis. Indeed, some women with endometriosis experience reduced levels of acetate and β‐hydroxybutyrate.^10^, ^76^ Conversely, progressive mitochondrial dysfunction can lead to a diminished role of fatty acid β‐oxidation and an increased reliance on glycolysis in overall energy metabolism.^74^ The shift between anaerobic glycolysis and lipid metabolism in different endometriosis phenotypes may depend on the degree of mitochondrial dysfunction.^74^

#### 3.2.3 Polycystic ovary syndrome (PCOS)

PCOS is the most common endocrine and metabolic disorder affecting women of reproductive age and is associated with insulin resistance, even in the absence of overweight.^22^, ^77^ Metabolite levels were analyzed and contrasted between normal‐weight women with PCOS and those without the condition. The green arrows in Figure 4 identify the metabolites altered in PCOS patients. An elevated concentration of glucose and reduced concentrations of lactate and pyruvate have been observed in the FF of PCOS patients (yellow square).^41^, ^64^, ^78^ This characteristic is attributed to altered metabolic pathways with decreased aerobic glycolysis in PCOS patients,^64^ which contrasts with findings in older women and those with endometriosis. High glucose and low lactate levels in FF have been linked to impaired oocyte maturation and pregnancy failure.^17^, ^41^ Moreover, alterations in glycerolipid and glycerophospholipid metabolic pathways have been identified in PCOS patients.^24^, ^79^ The levels of plasmalogens containing oleic acid, arachidonic acid, choline, or ethanolamine, as well as other lipids such as PA, LysoPA, LysoPE, and LysoPC, were significantly decreased in FF from women with PCOS (green square).^79^, ^80^ Plasmalogens and their metabolites (e.g., prostaglandins, leukotrienes, eicosatrienoic acid, dihydroxyeicosatetraenoic acid, eicosatetraenoic acid, and lipotoxins) are known for their antioxidant potential and immunomodulatory effects.^80^, ^81^ Consequently, plasmalogens may play a role in the pathophysiological processes of hypoxia, inflammation, oxidative stress, immunomodulation, and ferroptosis in the granulosa cells of PCOS patients.^80^ Indeed, decreased plasmalogen levels are associated with reduced oocyte quality.^80^ DHAP serves as a precursor for plasmalogen biosynthesis.^80^ Therefore, in PCOS patients, compromised glycolysis in granulosa cells may reduce the production of DHAP, subsequently impairing plasmalogen biosynthesis and resulting in lipid metabolism dysfunction. Additionally, PCOS patients are prone to glycolipid metabolism disorders, often linked to insulin resistance and dyslipidemia.^79^ Furthermore, carnitine levels have been reported to be significantly lower in women with PCOS.^79^, ^82^ Carnitine facilitates the transport of long‐chain fatty acids into mitochondria, where they are converted into energy.^79^, ^83^ In granulosa cells of women with PCOS, reduced carnitine levels contribute to impaired β‐oxidation and mitochondrial oxidative phosphorylation via acetyl‐CoA, leading to an energy deficit. Nevertheless, significant discrepancies exist in the literature concerning the levels of lipid metabolites.^64^

#### 3.2.4 Obesity

The black arrows in Figure 5 highlight the metabolites altered in obese women. Obesity leads to significant alterations in metabolites within the FF of women undergoing IVF, largely due to inflammation, oxidative stress, and disrupted lipid metabolism.^84^ Previous research has demonstrated that elevated glucose levels in the FF of obese women have been observed, potentially impairing oocyte function and influencing fertilization and embryo development.^41^, ^85^ Furthermore, the expression of genes in oocytes that regulate inflammation and oxidative stress (e.g., upregulation of aminomalonate, 2‐ketoglucose dimethylacetal, indole‐3‐propionic acid, and isothreonic acid) and lipid metabolism (e.g., upregulation of uric acid and downregulation of carnitine) undergoes considerable changes.^84^, ^86^, ^87^ The black and green arrows indicate similar metabolic trends (e.g., increased glucose, decreased lactate, decreased lipids, decreased carnitine, and increased purine degradation), suggesting that the metabolic patterns of obesity and PCOS are closely aligned.

#### 3.2.5 Clinical conditions leading to infertility

Several studies have compared the metabolic profiles of FF in patients with various clinical conditions associated with infertility, including endometriosis, PCOS, age‐related reduced ovarian reserve (AR‐ROR), reduced ovarian reserve (ROR), unexplained infertility (UI), and genetic infertility (GI).^88^ Patients with endometriosis, AR‐ROR, ROR, and UI exhibited lower glucose and higher lactate levels,^88^ indicating that in conditions other than PCOS, there is an elevation in glycolytic activity, accompanied by increased glucose consumption and lactate overproduction during oocyte maturation, leading to reduced mitochondrial oxidation. In comparisons between women who became pregnant following IVF and those who did not, the latter group showed lower lipid levels and altered lipid composition (e.g., PA, PE, PC, PI, glucosylceramides, and 1‐hydroxyvitamin D3 3‐D‐glucopyranoside).^2^ These metabolites play roles in cell proliferation, inflammation, signal transduction, and apoptosis, and may contribute to reduced oocyte quality.^2^ Additionally, the concentrations of hypoxanthine and xanthine were significantly elevated in the FF of the infertile group.^88^ Hypoxanthine, a degradation product of ATP, is generated during energy metabolism and is further converted into uric acid (gray square). Studies have shown that increases in hypoxanthine and xanthine indicate ATP depletion.^89^ Energy deficiency signifies a failure of compensatory mechanisms, ultimately leading to infertility and ART failure.

## 4 DISCUSSION

**FIGURE 6:**
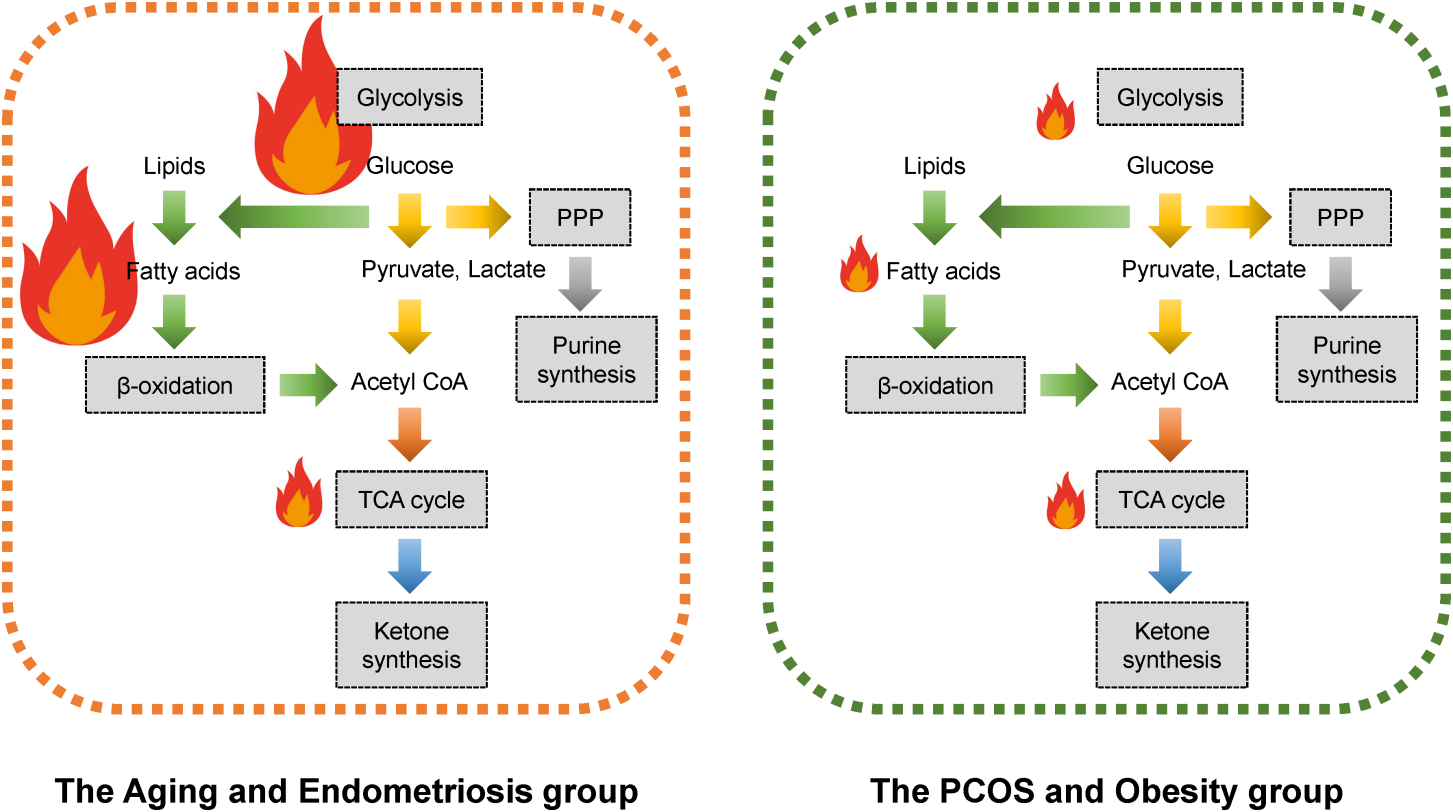
Anticipated metabolic pathways in women with infertility. The flame's size visually represents the energy level.

Infertility is a multifaceted condition influenced by various factors such as age, obesity, specific treatment modalities, and conditions like endometriosis and PCOS. Therefore, an ideal biomarker must be distinct and reliable, independent of confounding factors. The predicted metabolic pathways in women with several pathological conditions are visually represented in Figure 6. Several metabolites related to energy metabolism in infertile patients are significantly affected across five pathways: glycolysis, oxidative phosphorylation, lipid metabolism and β‐oxidation, nucleic acid synthesis, and ketogenesis. Additionally, metabolomic profiling may potentially classify these patients into two categories: infertility associated with aging and endometriosis, and infertility related to PCOS and obesity.

First, glycolysis and lipid metabolic profiles differ between the aging and endometriosis group and the PCOS and obesity group. In the aging and endometriosis group, glycolysis and lipid metabolism are upregulated to compensate for mitochondrial dysfunction, whereas the opposite trend is observed in the PCOS and obesity group.^64^ In the aging and endometriosis group, glycolysis and lipid metabolism synergize to enhance the FF environment and counteract adverse conditions such as inflammation, oxidative stress, and aging. Metabolomic analysis also revealed activation of nucleic acid synthesis and antioxidant signaling via the PPP. Conversely, in the PCOS and obesity group, lipid metabolism and β‐oxidation are often reduced due to diminished levels of fatty acids and carnitine. Although the glycolytic pathway supports the production of plasmalogens and fatty acids, further impairment in glycolysis and lipid metabolism could lead to irreversible damage to energy metabolism. Therefore, in the PCOS and obesity group, abnormal glycolysis and mitochondrial dysfunction may lead to a decreased number of high‐quality embryos.^90^ Ultimately, in both groups, mitochondrial dysfunction results in compromised oocyte quality due to inadequate energy production. Collectively, in the aging and endometriosis group, there may be an activation of glycolysis and lipid metabolism to compensate for the dysregulation of mitochondrial oxidative phosphorylation. Conversely, in the PCOS and obesity group, irreversible changes in glycolysis and lipid metabolism could contribute to mitochondrial dysfunction. It is plausible that the compensatory mechanisms of cumulus cells and oocytes are integrated through the interconnection of five energy metabolic pathways centered around acetyl‐CoA.

Second, fatty acids and glycolysis are intricately connected in cellular energy metabolism, adapting to the energy demands of the cell. In energy‐rich FF environments, glucose is metabolized through glycolysis, and fatty acids are stored, whereas during energy shortages, fatty acids are degraded to serve as fuel. In addition to their role as energy suppliers, fatty acids also participate in cellular signaling.^91^, ^92^ PA undergoes dephosphorylation to form DAG, an intermediate in glycerolipid metabolism that facilitates the activation of protein kinase C (PKC). DAG also plays a role in synthesizing prostaglandins, which are implicated in inflammatory responses, platelet aggregation, and several pathophysiological processes, including cell proliferation, oncogenesis, phagocytosis, and apoptosis.^91^, ^93^ PKC isotypes are crucial in various biological functions, including the resumption of meiosis in oocytes, spindle organization during meiosis, and chromosome movement.^91^, ^93^ Hence, the development and maturation of follicles, along with the quality of oocytes, may be reliant on energy metabolism and the quantity and composition of fatty acid metabolites within the follicular fluid.

Third, the findings of this metabolomic study, in conjunction with genomic, transcriptomic, and proteomic analyses, offer a deeper understanding of the interplay among energy metabolism, follicular development and maturation, and oocyte quality. For instance, endometriotic cells have developed a range of adaptive strategies to endure the chronic conditions of hypoxia and nutrient deprivation. Mitochondria serve as pivotal regulators, integrating various physiological processes such as energy production, cellular redox homeostasis, mitochondrial dynamics, and apoptosis regulation.^94^ To limit ROS generation, endometriotic cells favor glycolysis over oxidative phosphorylation and maintain mitochondrial quality control mechanisms through mitophagy and autophagy.^94^ This could lead to compromised oocyte development, maturation, and quality due to the downregulation of oxidative phosphorylation. Conversely, in patients with PCOS, hyperandrogenism triggers altered gene expression associated with glycolysis, mitochondrial biogenesis, mitochondrial fission and fusion dynamics, and mitophagy, culminating in abnormal mitochondrial morphology and disruption of the electron transport chain.^95^ Dysfunctional mitochondrial dynamics may undermine quality control mechanisms and impair the compensatory capacity of mitochondrial biogenesis, thereby further compromising mitochondrial function and diminishing oocyte quality.^95^ Thus, the phenotype and severity of infertility dictate the shift in energy metabolism from adaptive to compensatory and eventually to pathological conditions. This shift finely adjusts the equilibrium among glycolysis, oxidative phosphorylation, fatty acid synthesis, and β‐oxidation. Glycolysis and lipid metabolism vary greatly depending on whether compensatory mechanisms are still maintained or have already been compromised. In other words, IVF failure may occur along with the deterioration of effective compensatory mechanisms of energy metabolism.

Finally, we address current challenges and future research directions. It is often suggested that comprehensive metabolomics data analysis, combined with genomic, transcriptomic, and proteomic data, can help elucidate the mechanisms of IVF success or failure.^61^ In recent years, advancements in AI analysis technology have significantly enhanced the interpretation of complex omics data. However, in clinical practice, there is a need for molecular targets that can be easily and inexpensively tested at any time and place. It is worth measuring glucose, pyruvate, lactate, and plasmalogen levels in FF. The dynamics of these parameters may reflect mitochondrial activity required for oocyte growth and maturation. However, it is still premature to determine whether these metabolic changes can serve as reliable indicators of oocyte quality and ART success in clinical practice. Currently, there is no evidence to suggest that altering metabolomic profiles can enhance reproductive outcomes.^17^ Thus, while metabolomic analysis of FF may hold potential for predicting female reproductive outcomes, ideal biomarkers remain to be identified. Future research should urgently explore whether combinations of these biomarkers correlate with the number of oocytes retrieved, the quantity of mature oocytes (MII), and the generation of high‐quality embryos.

## CONFLICT OF INTEREST STATEMENT

The authors declare no conflict of interest.

## Data Availability

No new data were created.
